# Deep-Learning-Aided Evaluation of Spondylolysis Imaged with Ultrashort Echo Time Magnetic Resonance Imaging

**DOI:** 10.3390/s23188001

**Published:** 2023-09-21

**Authors:** Suraj Achar, Dosik Hwang, Tim Finkenstaedt, Vadim Malis, Won C. Bae

**Affiliations:** 1 Department of Family Medicine, University of California-San Diego, La Jolla, CA 92093, USA; 2 Department of Electrical and Electronic Engineering, Yonsei University, Seoul 03722, Republic of Korea; 3 Center for Healthcare Robotics, Korea Institute of Science and Technology, Seoul 02792, Republic of Korea; 4 Department of Radiology, Research Institute of Radiological Science and Center for Clinical Imaging Data Science, Yonsei University College of Medicine, Seoul 03722, Republic of Korea; 5 Department of Oral and Maxillofacial Radiology, Yonsei University College of Dentistry, Seoul 03722, Republic of Korea; 6 Institute of Diagnostic and Interventional Radiology, University Hospital Zurich, University Zurich, 8091 Zurich, Switzerland; 7 Department of Radiology, University of California-San Diego, La Jolla, CA 92093, USA; 8 Department of Radiology, VA San Diego Healthcare System, San Diego, CA 92161, USA

**Keywords:** lumbar spine, pars, low back pain, image processing, image regression, bone fracture

## Abstract

Isthmic spondylolysis results in fracture of pars interarticularis of the lumbar spine, found in as many as half of adolescent athletes with persistent low back pain. While computed tomography (CT) is the gold standard for the diagnosis of spondylolysis, the use of ionizing radiation near reproductive organs in young subjects is undesirable. While magnetic resonance imaging (MRI) is preferable, it has lowered sensitivity for detecting the condition. Recently, it has been shown that ultrashort echo time (UTE) MRI can provide markedly improved bone contrast compared to conventional MRI. To take UTE MRI further, we developed supervised deep learning tools to generate (1) CT-like images and (2) saliency maps of fracture probability from UTE MRI, using ex vivo preparation of cadaveric spines. We further compared quantitative metrics of the contrast-to-noise ratio (CNR), mean squared error (MSE), peak signal-to-noise ratio (PSNR), and structural similarity index (SSIM) between UTE MRI (inverted to make the appearance similar to CT) and CT and between CT-like images and CT. Qualitative results demonstrated the feasibility of successfully generating CT-like images from UTE MRI to provide easier interpretability for bone fractures thanks to improved image contrast and CNR. Quantitatively, the mean CNR of bone against defect-filled tissue was 35, 97, and 146 for UTE MRI, CT-like, and CT images, respectively, being significantly higher for CT-like than UTE MRI images. For the image similarity metrics using the CT image as the reference, CT-like images provided a significantly lower mean MSE (0.038 vs. 0.0528), higher mean PSNR (28.6 vs. 16.5), and higher SSIM (0.73 vs. 0.68) compared to UTE MRI images. Additionally, the saliency maps enabled quick detection of the location with probable pars fracture by providing visual cues to the reader. This proof-of-concept study is limited to the data from ex vivo samples, and additional work in human subjects with spondylolysis would be necessary to refine the models for clinical use. Nonetheless, this study shows that the utilization of UTE MRI and deep learning tools could be highly useful for the evaluation of isthmic spondylolysis.

## 1. Introduction

Low back pain (LBP) is common in pediatric and adolescent patients, particularly in athletes. Spondylolysis is defined as a bony defect in the pars interarticularis of the vertebral neural arch of the lumbar spine. In particular, isthmic spondylolysis [1], characterized by unilateral or bilateral bone stress injury secondary to repetitive mechanical spinal loading during activity [2,3], is found in as many as 50% of adolescent athletes with persistent LBP [2,4,5,6,7,8,9]. Failure to make the correct diagnosis may lead to a premature return to activities that can be detrimental to the healing of the spondylolysis.

For clinical evaluation of isthmic spondylolysis, a physical exam such as palpation of spinous processes, musculature around affected spinous processes [10], and the single leg extension test [11,12] may be used with a limited diagnostic benefit [13]. Imaging is also frequently performed. Plain radiography, the first line of imaging, has low sensitivity (~60%) [14]. Computed tomography (CT) is currently the reference standard for the detection of pars fractures, with sensitivity as high as 90% [15]. However, due to undesired radiation exposure (5–10 mSv) and the exposure of reproductive organs, particularly in young patients, the use of CT is being discouraged in favor of magnetic resonance imaging (MRI), which does not expose young athletes to ionizing radiation.

Conventional MRI is often prescribed if a plain X-ray and physical exam are inconclusive. It provides good soft tissue contrast and bone marrow changes that may be suggestive of an early isthmic spondylolysis [16,17], but it has low sensitivity for detecting bony defects, and inconclusive diagnosis will require an additional CT scan. In recent studies where CT was used as the reference standard, the sensitivity of conventional sagittal and axial MRI for the diagnosis of spondylolysis was 73–92% for a variety of pars fractures [18,19,20,21], suggesting ~20% lower sensitivity compared to a CT exam [16,17,22]. Efforts have been made to improve sensitivity by optimizing imaging planes and trying various axial and sagittal oblique planes [23], as well as utilizing fat-suppressed and fluid-sensitive sequences that sometimes reveal signals from the scar tissues filling the pars defect [24]. But these add complexity and time and do not always improve the diagnosis. The main reason for the lower sensitivity of conventional MR protocols includes short T2 properties of the cortical bone and the osteofibrous scar tissues near the pars defect, whose signals decay rapidly [25,26] in conventional MR sequences using echo times (TE) greater than 10 ms. As a result, there is very little contrast between the cortical bone and the pars defect, which hinders the diagnosis.

Recently, we have shown that a newer technique known as ultrashort time-to-echo (UTE) MRI depicts the osseous components of the lumbar spine with higher contrast than conventional MRI and improves the detection of experimental spondylolysis [27]. UTE-based MRI techniques can utilize TE of 1 ms or less [28,29,30,31,32,33,34,35,36,37,38,39] and acquire sufficient MR signal from the scar tissue to detect spondylolysis. UTE MRI can depict cortical bone with a uniform contrast (i.e., low signal for the bone and the air and high signal for all other tissues), making it relatively easy to isolate and visualize the bone, with conventional image processing. Past studies have also shown that cortical bone morphology (e.g., surface contour) measured on UTE MRI is similar to that measured on CT [38,40].

While a marked advancement, even UTE MRI images have undesirable characteristics that may hinder the detection of spondylolysis. These include uneven shading from posterior surface coils, varying signal intensities from the soft tissues, and relatively low contrast for bone compared to CT. Additionally, general radiologists are not familiar with UTE MRI images, as these are relatively new and are only beginning to become available on many MRI scanners. On the other hand, radiologists are very familiar with spine CT, and it is relatively fast and easy to identify pars defects on CT images. It would be clinically impactful if tools became available to generate CT-like images from MRI images and to provide suggestions for the locations of pars defects.

We believe deep learning models could be useful in this regard. Deep learning models have been proposed for generating pseudo-CT or CT-like images from MRI, particularly for attenuation correction for PET/MR setting [41,42,43,44], as well as for cranial bone imaging [45,46,47] during stereotactic radiotherapy. Using a single modality can shorten treatment planning time and reduce the risk of misregistration. Several different deep learning techniques have been proposed to achieve CT-like synthesis from MRI data [48]. These included atlas-based and voxel-based techniques. Atlas-based techniques [49,50] use pairs of MRI and CT atlas that are averaged from multiple datasets. When new MRI data are obtained, the MRI atlas is deformed to match the data, and the deformation is applied to the CT atlas to create the CT-like image. Created for dosimetry purposes, atlas-based techniques introduce significant error for the personalized assessment of bone morphology. For voxel-based techniques, conversion of MRI voxel signal intensity to Hounsfield units (HU) at different anatomic regions using simple [51] or complex regression models [52] have been used. Deep learning approaches including U-Net [53] and generative adversarial network (GAN) [46,53] have also been introduced, showing good results in predicting the dose needed for radiotherapy.

However, little attention has been given to the generation of CT-like images for diagnostic purposes. Additionally, while bone fracture detection in medical images using deep learning has been an ongoing area of research [54], existing techniques may be suboptimal in terms of 3D visualization of the regions with high fracture probability. We hope to expand on past work while focusing on spinal bone imaging, generating sharp and high-contrast CT-like images from UTE MRI images, and fracture maps to aid in the diagnosis and the detection of the injured area. As mentioned above, no attempt has been made to create CT-like images for the spine using deep learning, a region with complex bone anatomy, or a dedicated network for the detection of spondylolysis. While we will utilize networks that have been described previously, the application to this anatomy and disease is novel with a significant clinical benefit.

Expanding upon our past study on UTE MRI of the lumbar spine spondylolysis, the purpose of this proof-of-concept study was to develop deep learning U-Net-based tools that read in UTE MRI images of cadaveric lumbar spines and output (1) CT-like images for easier interpretation and (2) a saliency map of fracture probability for quick detection of pars fracture. It is hoped that these experimental tools may be refined and translated in the future for the clinical evaluation of isthmic spondylolysis.

## 2. Materials and Methods

This study involved the use of de-identified MRI images of cadaveric specimens and was exempted by the institutional review board.

### 2.1. Imaging Data 

**Figure 1:**
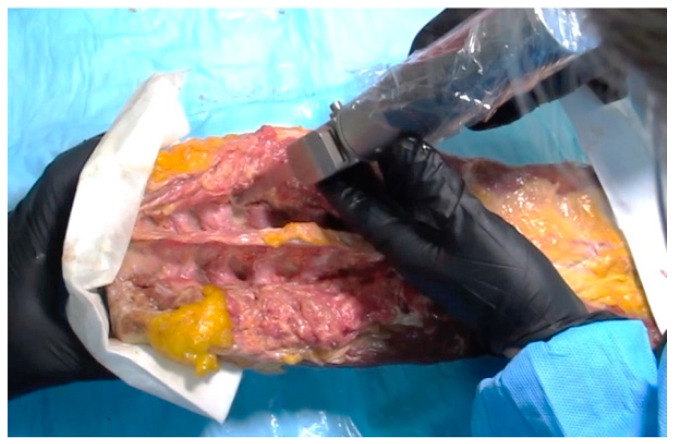
Experimental pars defect being created on a cadaveric lumbar spine with a bone saw.

**Figure 2:**
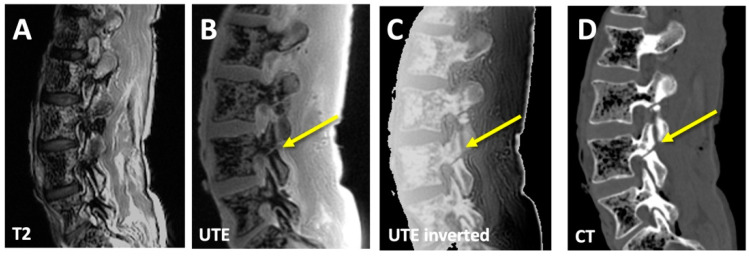
Right-sided imaging of a cadaveric spine, showing typical sagittal images of an experimental pars fracture at L4. On the conventional T2 images, (**A**) the experimental pars defect on the right L5 level is not visible. In contrast, raw (**B**) and inverted (**C**) UTE images can depict the fracture (arrow), albeit with a lower contrast compared to the CT image (**D**).

In our previous study [27], we obtained cadaveric lumbar spines (*n* = 4; 3 female, 1 male, 54 ± 18 years) en bloc, with muscles intact. We requested samples without previous history of surgery to avoid complications with possible instrumentation. No other selection criteria were used. At randomly chosen sites of pars interarticularis (*n* = 20 sites), bone fractures were created with a surgical saw to simulate spondylolysis (Figure 1). Specimens were scanned on a 3-Tesla MRI scanner (General Electric Healthcare, Discovery 750) in the sagittal plane with conventional SE T2 (Figure 2A; TR = 4600 ms, TE = 102 ms, matrix = 224 × 224, slice = 3 mm, FOV = 24 cm, time = 2:14 min) and 3D UTE (Figure 2B; TR = 40 ms, TE = 0.03 ms, number of spokes = 6000; matrix = 256 × 256, slice = 2 mm, FOV = 24 cm, FA = 2 deg, time = 4:00 min). These parameters were chosen to mimic those used in clinical settings, with similar spatial resolution and scan time.

Computed tomography (CT) scans (Figure 2D) were performed on a 256-MDCT scanner (Revolution; General Electric Healthcare, Chicago, IL, USA): 120 kV, 100 mA, slice = 0.625 mm, reconstruction diameter = 50 cm, and matrix = 512 × 512.

Image registration: The MRI and CT images were stacked to create individual 3D volumes for each specimen and rescaled to have the same voxel size of (0.625 mm)^3^. Three-dimensional rigid body image registration was performed using FMRIB’s linear image registration tool (FLIRT) [55] using only translation and rotation. The co-registered images were cropped in-plane to 224 × 384 voxels while leaving the number of slices unmodified. The images were then augmented via translation, rotation, rescaling, and the combination of all three while keeping the final cropped image size of 224 × 384. After augmentation of 509 image pairs, a total of 2545 training image pairs were available for training. In this proof-of-concept study to demonstrate feasibility and utility, due to the limited available data, we used all datasets for training and for results. No separate testing data were used.

### 2.2. Deep Learning: Image Regression Model

**Figure 3:**
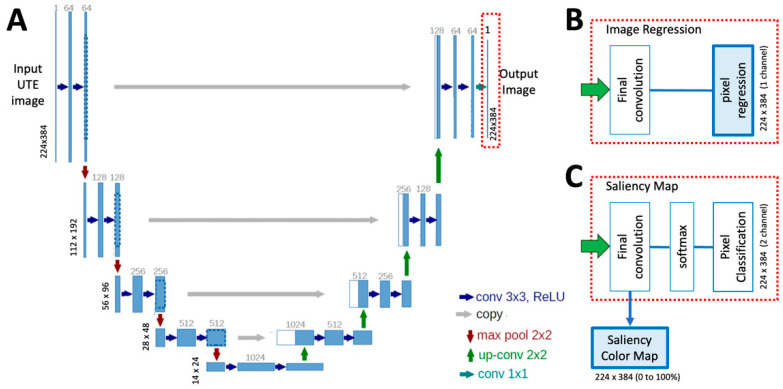
Architecture of a standard U-Net model (**A**), which was modified (red box) for (**A**) image regression (**B**) and saliency mapping (**C**). The final output images are shown in blue-filled boxes.

**Figure 4:**
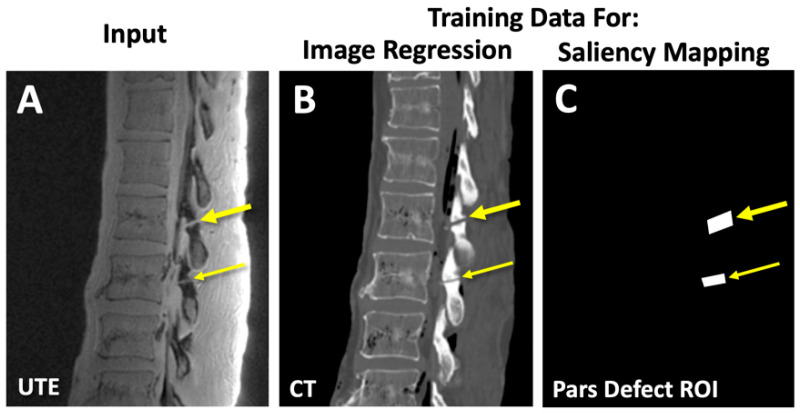
Deep learning model training. We developed an image regression deep learning model that takes (**A**) UTE MRI images of the lumbar spine as an input and compared against (**B**) registered CT images as the ground truth. Saliency mapping model also takes (**A**) UTE MRI images as the input and compared against (**C**) annotations of pars defect regions of interest (ROI) as the ground truth.

We built a convolutional neural network (CNN) based on 2D U-Net architecture (Figure 3A) [56] modified (Figure 3B) to perform a supervised image-to-image regression. The 2D U-Net was chosen based on its generally robust performance under many applications and our familiarity with the model from past studies. The model was written in Matlab with Deep Learning Toolbox (R2022b) and trained on a Windows computer with an RTX3090 GPU. UTE MRI images were normalized and used as the input (Figure 4A). Corresponding CT images (Figure 4B) were used as the ground truth for the supervised learning. The image regression model has 64 convolution filters with a 3 × 3 kernel size, 2 convolution operations at each step, with an encoder depth of 5 (Figure 3A). The final output layer (Figure 3B) is an image regression layer instead of a pixel classification typically used for U-Net image segmentation. The model was trained to 100 epochs using the default setting (Adam optimizer, root mean squared error loss function, shuffling image every epoch, and mini batch size of 8). We provide a pseudocode below in Section Matlab Code for Image Regression.

#### Matlab Code for Image Regression

% data directories
imageDir = “directory for MRI images”
labelDir = “directory for CT images”

% create image and label datastores
imds = imageDatastore(imageDir)
pxds = imageDatastore(labelDir)

% combined training datastore
dsTrain = combine(imds,pxds)

% create the baseline U-net
imageSize = [384 224]
encoderDepth = 5;
network1 = unetLayers(imageSize,2,‘EncoderDepth’,encoderDepth)

% create 2D convolution and regression layers to replace base Unet
layer_conv = “2D convolution layer with 1 channel output”
layer_reg = “regression layer with 1 channel output”

% remove softmax and segmentation layers and replace with regression layer
network2 = “command to remove segmentation layer from network1”
network2 = “command to replace final 2D convolution layer with layer_conv”
network2 = “command to replace softmax layer with layer_reg”

% change options to match your PC hardware
train_options = trainingOptions(‘adam’,…
‘LearnRateDropFactor’,0.05, …
‘LearnRateDropPeriod’,5, …
‘Shuffle’,’every-epoch’,…
‘MaxEpochs’,100, …
‘MiniBatchSize’,8); %, …

% start training
Regression_Network = trainNetwork (dsTrain, network2, train_options)

%%%%%%%% inference post training %%%%%%%%
CT_like_image = predict(Trained_Network, input_MRI)

### 2.3. Deep Learning: Saliency Mapping Model

Another CNN model was built for pars defect detection. The purpose of this model was to provide visual cues to help the reader locate the problem area of the spine. We used the same architecture as the image regression model, except at the output end, we used a softmax activation followed by a pixel classification layer instead of a regression layer. For training, augmented UTE MRI images (Figure 4A) were annotated using ImageJ by segmenting regions of pars fractures as binary images (Figure 4C). After training, we visualized the activation or “saliency map” of the final convolution layer prior to softmax activation (Figure 3C), which contains the probability of the presence of the pars defect (Figure 4C). This model was also trained to 100 epochs using the default setting (Adam optimizer, cross-entropy loss function, shuffling image every epoch, and mini batch size of 8).

#### Matlab Code for Saliency Mapping

% data directories
imageDir = “directory for MRI images”
labelDir = “directory for annotations for pars defects”

% create image and label datastores
imds = imageDatastore(imageDir)
pxds = imageDatastore(labelDir)

% combined training datastore
dsTrain = combine(imds,pxds)

% create the baseline U-net
imageSize = [384 224]
encoderDepth = 5;
network1 = unetLayers(imageSize,2,‘EncoderDepth’,encoderDepth)

% add class weights since the volume of defect is very small
tbl = countEachLabel(pxds);
numberPixels = tbl.ImagePixelCount;
frequency = tbl.PixelCount ./ numberPixels;
classWeights = 1 ./ frequency;

% replace the last layer with a weighted classification layer
layer1 = “segmentation layer with weighted class weights”
network2 = “command to replace the last segmentation layer with layer1”

% change options to match your PC hardware
train_options = trainingOptions(‘adam’,…
‘InitialLearnRate’,1e-5,…
‘Shuffle’,’every-epoch’,…
‘Verbose’,true,…
‘MaxEpochs’,100, …
‘MiniBatchSize’,8);

% start training
Saliency_Network = trainNetwork (dsTrain, network2, train_options)

%%%%%%%% saliency mapping post training %%%%%%%%

testimg = imread(“test image”);
act = activations(net1,testimg,’Softmax-Layer’); % “see” the activation layer
figure
imagesc(act(:,:,2))

### 2.4. Outcome Measures

#### 2.4.1. Similarity Measures between CT and CT-like Images

To compare the quantitative similarity of (i) inverted UTE (Figure 2C) and CT images and (ii) CT-like and CT images, we determined the mean squared error (MSE; Equation (1)) [57] and peak signal-to-noise ratio (PSNR; Equation (2)) [57] for each image pair (*n* = 509) as follows:

(1) $$MSE=\frac{\sum {\left({SI}_{i}^{CTlike}-{SI}_{i}^{CT}\right)}^{2}}{n}$$

(2) $$PSNR=10\times {log}_{10}\left(\frac{255^{2}}{MSE}\right)$$

where ${SI}_{i}^{CT\;like}$ is the signal intensity of an *i*-th voxel in a CT-like image, ${SI}_{i}^{CT}$ is the signal intensity of an *i*-th voxel in a CT image, and *n* is the number of voxels in an image (i.e., 224 × 384 = 86,016 voxels). The values were then averaged for all of the data.

We also determined the structural similarity index (SSIM; Equation (3)) [58], an image quality metric that compares the visual characteristics of the luminance, contrast, and structure between images *x* and *y* (e.g., CT and CT-like images, respectively).

(3) $$SSIM\left(x,y\right)=\frac{\left(2\mu_{x}\mu_{y}+c_{1}\right)\left(2\sigma_{xy}+c_{2}\right)}{\left({\mu_{x}^{2}}_{}+{\mu_{y}^{2}}_{}+c_{1}\right)\left({\sigma_{x}^{2}}_{}+{\sigma_{y}^{2}}_{}+c_{2}\right)}$$

where $\mu_{x}$ and $\mu_{y}$ are the pixel sample means of image *x* and *y*, respectively; $\sigma_{xy}$ is the cross-correlation of *x* and *y*; $\sigma_{x}$ and $\sigma_{y}$ are the variance of *x* and *y*; $c_{1}={\left(k_{1}L\right)}^{2}$, where $k_{1}$ = 0.01 and *L* is the dynamic range (i.e., 2^8^ − 1 = 255 for 8-bit grayscale images); and $c_{2}={\left(k_{2}L\right)}^{2}$, where $k_{2}$ = 0.03.

MSE is a traditional metric, and it measures the squared difference between the pixel values of two images. PSNR is also a widely used metric, and it shows a ratio between the maximum possible power of a signal and the power of the noise. While these metrics are great for a perfectly registered set of images, they do not consider the structure of the image. SSIM considers the perception of the human visual system, and it models image distortion as a combination of factors that affect human perception. Since all three metrics are commonly used in reporting image fidelity and similarity, we determined all of them in this study.

#### 2.4.2. Contrast-to-Noise Ratio (CNR)

**Figure 5:**
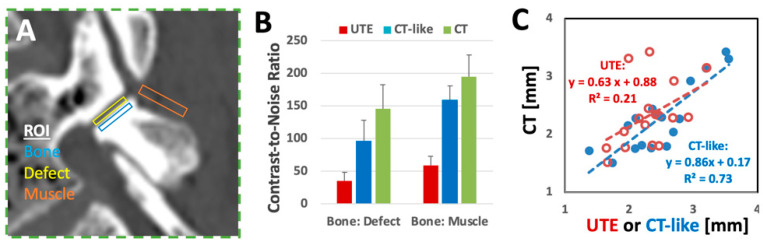
Contrast-to-noise ratios (CNRs) were measured on UTE, CT-like, and CT images of experimental pars defects created on three cadaveric spines. (**A**) Regions of interest for measuring the mean signal intensity of the bone, pars defect, and paraspinal muscles. (**B**) CNR of bone vs. pars defect and bone vs. surrounding muscles suggested the lowest CNR for UTE images compared to CT-like and CT images. (**C**) Width of pars defect was measured on UTE and CT-like images and correlated against the measurements on the reference CT images.

To compare image contrast between the UTE, CT-like, and CT images, we measured the mean signal intensity of the bone, pars defect, and paraspinal muscles using regions of interest (ROIs) (Figure 5A) at *n* = 11 pars defects. The standard deviation of the background noise signal intensity was also measured. CNR was determined (Figure 5B) as the difference between the mean signal intensity (*SI_mean_*) of two ROIs divided by the standard deviation (*SI_SD_*) of the noise as follows (Equation (4)) [59]:

(4) $${CNR}^{1-2}=\frac{{SI}_{mean}^{1}{-SI}_{mean}^{2}}{{SI}_{SD}^{noise}}$$

#### 2.4.3. Width Measurement of Pars Defects

Additionally, at randomly selected pars defects (*n* = 11), we measured the width of the defects on registered UTE, CT-like, and CT images. Using the measurement on CT as the reference, we plotted the width measurements on CT vs. UTE and CT vs. CT-like images (Figure 5C).

### 2.5. Statistics

The values of MSE, PSNR, and SSIM involving inverted UTE and CT-like images were compared using a *t*-test. CNR values involving UTE, CT-like, and CT images were compared using ANOVA with the post hoc Tukey test. Correlations between defect widths measured on CT vs. UTE and CT vs. CT-like images were determined using Pearson correlation and linear regression. 

## 3. Results

### 3.1. CT-like Images

Compared to conventional MRI such as spin echo T2 (Figure 2A), UTE images depicted spinal bone distinctly (Figure 2B), and when inverted (Figure 2C), had a similar appearance to a CT image (Figure 2D). Even so, inverted UTE images had uneven shading from posterior surface coils, varying signal intensities from the soft tissues, gas/air inside the vertebral body being depicted with high signal intensity, and a relatively low contrast for bone, making them a less than perfect surrogate for CT images.

**Figure 6:**
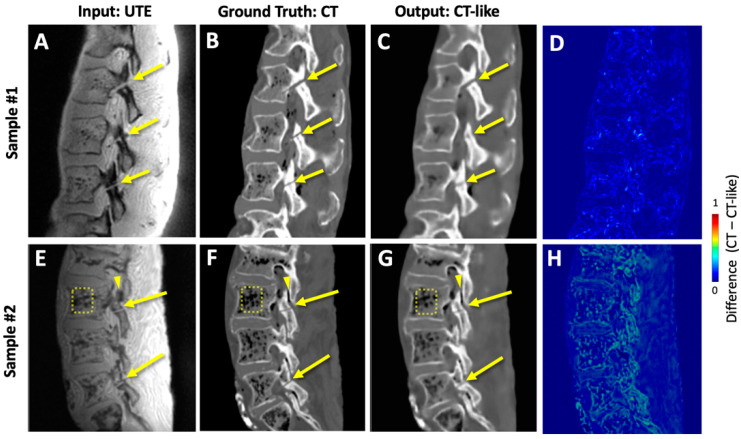
Results of image regression. UTE images (**A**,**E**) were used as input and trained on CT images (**B**,**F**) to synthesize CT-like images (**C**,**G**). Difference between CT and CT-like images is shown as color maps (**D**,**H**). Arrows indicate pars defects created experimentally. Compared to (**E**) UTE image that depicted both the bone and air in the vertebral body (square) and the facet joint (arrowhead) with low signal intensity, (**F**) CT-like image correctly depicted the bony structures with high signal intensity and the air with low signal intensity. The overall correspondence between CT-like and CT images was excellent, but CT-like images were not as sharp.

**Figure 7:**
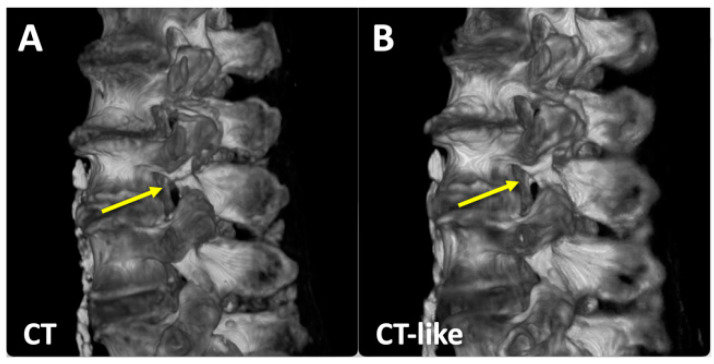
Three-dimensional bone renders of (**A**) CT and (**B**) CT-like datasets of a cadaveric spine showing the pars defect (arrow).

CT-like images generated by our deep learning network addressed many of the limitations of the UTE MRI images. Compared to UTE MRI images that had uneven shading (Figure 6A,E), our deep learning generated CT-like images provided even signal intensity of soft tissues on the posterior side (Figure 6C,G). UTE MRI images depicted both the bone and the air in the vertebral body (Figure 6E, square) and the facet joint (Figure 6E, arrowhead) with a low signal intensity, while CT-like images correctly depicted the bone with high signal intensity and the air with low signal intensity (Figure 6G). The correspondence between the ground truth CT (Figure 6B,F) and CT-like (Figure 6C,G) images was excellent, but the CT-like images were visibly softer. Color maps showing the difference between CT and CT-like images (Figure 6D,H) suggested a good fitting during the supervised learning. The CT-like image also facilitated 3D rendering and visualization: the 3D bone renders of the CT (Figure 7A) and CT-like (Figure 7B) images were nearly identical, and the pars fractures were obvious on both renders (arrows).

Table 1 compares quantitative measures of image similarity between (1) inverted UTE and CT images and (2) CT-like and CT images. All measures suggested greater dissimilarity and fidelity of the inverted UTE images than CT-like images when compared to the reference CT image. MSE of inverted UTE vs. CT (0.0528 ± 0.0340) was over 10 times greater than that of CT-like vs. CT (*p* = 2.6 × 10^−133^). PSNR of inverted UTE vs. CT (16.5 ± 14.2 dB) was significantly lower (*p* = 6.6 × 10^−53^) than CT-like vs. CT (28.6 ± 6.1 dB), and SSIM of UTE vs. CT (0.68 ± 0.19) was also lower (*p* = 0.0012) than that of CT-like vs. CT (0.73 ± 0.28).

For the CNR of bone vs. defect (Figure 5B), UTE had the lowest value at ~35 ± 14 (mean ± SD), followed by CT-like at 97 ± 31 and CT at 146 ± 37. These differences were statistically significant (post hoc *p* < 0.001 each). Trends were similar for the CNR of bone vs. muscle.

The width of the defect measured on CT-like images matched more closely to those measured on CT images. Linear regression of defect width on CT vs. CT-like images had a slope of 0.86 (near 1.0) and R^2^ = 0.73. In contrast, CT vs. UTE had a slope of 0.63 with a low R^2^ = 0.21.

### 3.2. Saliency Mapping

**Figure 8:**
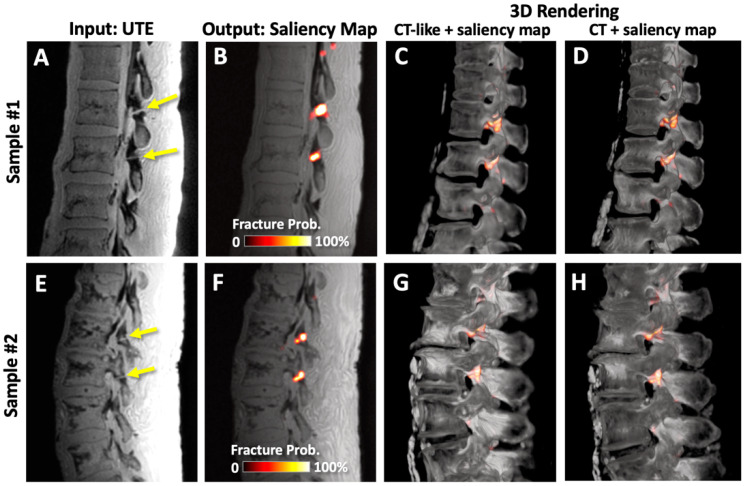
Saliency mapping model takes in (**A**,**E**) UTE images and outputs saliency colormaps. (**B**,**F**) Saliency maps overlaid onto UTE images show colored areas covering the pars defects. Saliency maps can also be fused with (**C**,**G**) CT-like images to create 3D fused rendering that highlights pars defects. Note the high similarity between the fused rending using (**C**,**G**) CT-like vs. (**D**,**H**) CT images.

Figure 8 shows the results of saliency mapping. We performed supervised learning using UTE images as the input (Figure 8A,E) and annotated regions of pars defect as the ground truth, but instead of performing segmentation, class activation of the final convolution layer was taken as the saliency heat map. Figure 8B,F shows heat maps fused with the UTE image, and the colors indicate the probability of the presence of the pars defect. When viewed side-by-side, this makes it quick and easy to detect and evaluate the pars defect. 

Combining saliency maps with CT-like images provides additional benefits. As a demonstration, we created a fused 3D render of CT-like data with the saliency map (Figure 8C,G), which could be useful for volumetric viewing and evaluation of the spinal bones. Note the visual similarity between the 3D renders using CT-like (Figure 8C,G) and CT (Figure 8D,H) images overlaid with fracture probability.

## 4. Discussion

We have shown a proof-of-concept study for implementing deep learning models to process UTE MRI images of human lumbar spines and to synthesize CT-like images as well as fracture probability heat maps. Additionally, we demonstrated the potential advantages of CT-like images over UTE images, including visual improvement and ease of evaluating the bone, greater similarity measures to reference CT images, and higher contrast-to-noise ratio. Saliency maps, when fused with UTE or CT-like images, provided additional improvement in making it quick and easy to detect and evaluate the pars defect.

Compared to UTE images, CT-like images provided multiple advantages for bone defect evaluation. First is the higher contrast-to-noise ratio for key regions of interest (i.e., cortical bone of pars interarticularis and scar tissue filling the pars defect). Reference CT images provide a CNR value averaging ~150, while UTE images had a mean CNR value of ~35. Our CT-like images had only a slightly lower (albeit statistically significant) mean CNR value near ~100. PSNR is a measure of image quality preservation between the original and a degraded image. PSNR for CT-like images was ~29, which is considered good, while for UTE images, PSNR was ~17, below the “acceptable” range [60]. 

While there have been many studies on deep learning models to create pseudo-CT images from MRI, most of them focused on dosimetry for radiotherapy applications. Relatively few studies exist with the goal of synthesizing images for diagnostic purposes. While our study uses a relatively established and popular U-Net model [61,62], other approaches exist, including a fully connected convolutional neural network (CNN) [63,64] and a generative adversarial network (GAN) [63,64,65]. Our model, despite being trained on a very limited dataset, provided a decent performance comparable to other models with PSNR in the range of 24 to 28 dB [62,63,64,66,67] and SSIM in the range of 0.65 to 0.79 [61,64,67]. While not performed in this study, feature extraction using deep learning [68] may be considered for the detection of pars defects, and the use of other models such as GAN may provide advantages when the relationship between the source and output images is difficult to establish (e.g., regression of conventional MRI images to CT images may perform better with GAN than CNN). 

This study has important clinical implications. Every year, many lumbar CT scans are performed on young athletes. Adding to this, the low back is one of the most frequently re-injured anatomy in sports. These factors can lead to a large number of repeated CT scans in young subjects, which is undesirable in terms of cancer risk as well as potential genetic risk for the next generation. If successful, a combination of MRI and deep learning processing to generate CT-like images can alleviate these issues. Additionally, the costs involved with receiving both MRI and CT can be reduced. Additionally, a deep learning aid such as that shown in Figure 8 to suggest the probable location of pars defects can be helpful for a faster and more accurate diagnosis while leaving the power of decision with the reader. Finally, the tools developed here can be utilized for any other diseases and injuries involving bone that currently require a CT scan to evaluate bone morphology.

This early study has several limitations. First, the size of our dataset was very limited, and all of the data were used for both training and testing. The data were “opportunistic” data from a previous study that was taken advantage of to perform this proof-of-concept study. Additionally, we used only UTE MRI images, and the applicability of the model with other types of MRI images (particularly clinical spin echo images that are more prevalent) is not yet certain. Only sagittal images were used, so additional training on other planes or developing a 3D deep learning model would be also needed. The training data are dependent on the accurate registration of image pairs, which is not always achievable. Most of all, only cadaveric sample images were used, and retraining would be necessary with actual human subjects with and without spondylolysis. The same issue applies to saliency mapping; while found to be useful in quickly drawing attention to the problem area, it has not been validated in real patients with spondylolysis. In the future, we wish to address the above limitations by expanding the work to include additional types of MRI images in different planes, obtaining images from live human subjects in vivo, and obtaining data from patients confirmed with spondylolysis. Taking it even further, our technique may be used to study a large population of subjects suspected of the condition.

## 5. Conclusions

In conclusion, this study shows a proof-of-concept development and utility of deep learning tools to synthesize (1) CT-like images and (2) a saliency map of fracture probability. We showed quantitatively and qualitatively that CT-like images can closely mimic the reference CT images, and this may address many of the shortcomings of UTE MRI that hinder the evaluation of isthmic spondylolysis. Taking it a step further, combining CT-like images with a saliency map made it even easier to quickly identify pars defects. It is hoped that this concept for deep-learning-based tools may be refined and translated in the future for clinical evaluation of isthmic spondylolysis.

## Figures and Tables

**Table 1:** Similarity metrics between inverted UTE vs. CT and CT-like vs. CT.

	Inverted UTE vs. CT	CT-like vs. CT	*p*-Value
MSE	0.0528 ± 0.0340	0.0038 ± 0.0054	2.60 × 10^−133^
PSNR (dB)	16.5 ± 14.2	28.6 ± 6.1	6.60 × 10^−53^
SSIM	0.68 ± 0.19	0.73 ± 0.28	0.0012

## Data Availability

The data are not publicly available due to privacy issues.
